# Perception and Attitude of Pakistani Doctors Toward the Use of Telemedicine Technology

**DOI:** 10.7759/cureus.31556

**Published:** 2022-11-15

**Authors:** Nisha Zahid, Akhtar Ali, Babris Gul, Syed H Danish, Syeda N Israr, Junaid Anwar

**Affiliations:** 1 Department of Pharmacology and Toxicology, Sapienza University of Rome, Rome, ITA; 2 Department of Pharmacology, Ziauddin Medical College, Ziauddin University, Karachi, PAK; 3 Department of Healthcare Management, Ziauddin Medical College, Ziauddin University, Karachi, PAK; 4 Department of Community Health Sciences, Ziauddin Medical College, Ziauddin University, Karachi, PAK; 5 Department of Dental Material, Baqai Dental College, Baqai Medical University, Karachi, PAK; 6 Department of Pathology, Ziauddin Medical College, Ziauddin University, Karachi, PAK

**Keywords:** doctors, pakistani, attitude, perception, telemedicine

## Abstract

Background: Healthcare providers may improve healthcare delivery and make it available to more people by combining advanced technologies with high-quality network services. In this regard, telemedicine has improved healthcare providers' ability to provide services for a large number of individuals without their physical presence. Pakistan's healthcare system is a mix of government infrastructure, parastatal healthcare, the private sector, civil society, and charitable contributions. However, unawareness, poor education, lack of skills, lack of resources, and technical issues including internet connectivity, and load shedding have limited the accessibility of the Pakistani population towards advancement in the healthcare industry.

Methodology: It was a cross-sectional study conducted at a private tertiary care hospital from April to June 2022 in Karachi. To recruit the study participants, a purposive sampling technique was used. Practicing doctors (n=100), who were aware of telemedicine technology and agreed to participate in the study, were included as study participants. A self-developed proforma was administered to doctors to assess the study's objectives.

Results: Out of 100, 64% (n=64) of the participants have no experience working in telemedicine while only 36% have worked with telemedicine for at least one year. When asked about the application of telemedicine technology only 46% of the doctors knew the technology used. While only 42% of the doctors were familiar with the telemedicine tools, including a virtual stethoscope, pulse oximeter, etc. views regarding continue using telemedicine, 26% of the participants having no experience with telemedicine agreed that they would like to use telemedicine, 35% agreed to use telemedicine with improvement whereas 3% of them do not want to use the telemedicine technology.

Conclusion: The participants of the current study were aware of telemedicine technology and its advantages. However, despite their agreement, the majority of the doctors emphasized the improvement of the system to deliver better services by using this technology.

## Introduction

Healthcare providers may improve healthcare delivery and make it available to more people by combining advanced technologies with high-quality network services. In this regard, telemedicine is a more advantageous technology that can assist people to seek preventive therapy and improve their long-term health. This is especially true for those who are unable to receive adequate care due to financial or geographical constraints [1]. Telemedicine has developed as a method for overcoming healthcare system issues, and it is anticipated to become more feasible as information and communication technology advance and become more user-friendly [2]. Telemedicine has become an important entry point into the process of diagnosis, triage, and treatment as a result of the COVID-19 pandemic, in order to limit patient displacement to hospitals, allocate hospital space, and slow the spread of the disease. While telemedicine and other healthcare technologies have great potential, their use has the potential to exacerbate disparities due to the “digital divide,” which refers to the disparate access to and use of technology and the internet among communities and populations of various races/ethnicity and socioeconomic demographics due to social, language, financial, and other barriers [3,4].

Telemedicine has improved healthcare providers' ability to provide services for a large number of individuals without their physical presence. It has proven its worth in the pandemic and now after COVID-19, hospitals have initiated the way forward to get benefits from this technology. Although it started with video conferencing with every passing day the technology is getting more advanced, and the next generation of telemedicine technologies would have much more to offer [5]. Globally, more hospitals are looking at the benefits of telemedicine as a result of rising healthcare expenditures and a demand for better treatment. They want better communication between doctors and patients who live far away, as well as more efficient use of healthcare facilities. Telemedicine has also improved connectivity, resulting in fewer hospital readmissions and patients following their prescription care regimens in the latter [6]. The introduction of telemedicine technology has imposed positive changes in serving patients in the neighboring countries of Pakistan [7,8]. The greater contact advantage of telemedicine also applies to doctor-to-doctor communication. Doctors can use telemedicine to create support networks, trade skills, and improve healthcare services. Telemedicine is a method of providing medical care over the internet, typically via video chat. Both patients and healthcare providers will benefit from this technology. Telemedicine can enrich and enhance the overall patient experience, despite technical challenges and detractors [9].

Telemedicine has the potential to connect patients in even the most remote locations with trained doctors in urban areas. The rapid growth of the population, combined with an unstructured healthcare system, has resulted in an unequal distribution of doctors, resulting in a chronic doctor shortage in peri-urban and rural areas [10]. Pakistan's healthcare system is a mix of government infrastructure, parastatal healthcare, the private sector, civil society, and charitable contributions. Furthermore, the physician-to-population ratio in Pakistan is significantly lower, i.e., 1:1,127, than the WHO-recommended ratio of 1:1,000 [11]. However, unawareness, poor education, lack of skills, lack of resources, and technical issues including internet connectivity, and load shedding have limited the accessibility of the Pakistani population towards advancement in the healthcare industry. Hence, the study was designed to assess the attitudes of doctors towards acceptance of telemedicine technology and hindrances in its implementation so that intervention can be made to enter into the era of advanced healthcare service.

## Materials and methods

It was a cross-sectional study conducted at a private tertiary care hospital from April to June 2022 in Karachi. This article was approved by Ethics Review Committee of Ziauddin University (4210941AAPHA).

Sample selection criteria

The sample size was calculated by the open-epi sample size calculator. At 50% proportion of the total number of doctors in the tertiary care setup at a 95% confidence interval, the sample size was n=43; however, to overcome the data errors and to exclude unfilled or half-filled proforma 138 doctors were approached and completely filled data of n=100 was analyzed. To recruit the study participants, a purposive sampling technique was used. Practicing doctors, who were aware of telemedicine technology and agreed to participate in the study were included as study participants. Postgraduate students and other healthcare staff were excluded from the study.

Data collection procedure

A self-developed proforma highlighting the advantages, disadvantages, strengths, and limitations of telemedicine was administered to doctors to assess the study's objectives. Prior to the administration of the questionnaire, the study participants were given a brief introduction to the project and the objectives of the study. Doctors who agreed to participate and were according to the set inclusion criteria of the study were asked to fill out a written consent form. Data were collected and scrutinizing of the proforma was performed to select the completely filled form. Incomplete forms and forms with multiple item selection by the participant were excluded.

Data analysis

Quantitative data were analyzed by using SPSS version 22 (IBM Corp., Armonk, NY). Frequencies and percentages of responses against questions were calculated and chi-square analyses were applied as a test of significance at a 95% confidence interval, 5% margin of error, and a p-value less than 0.05 was considered as significant.

## Results

**Figure 1:**
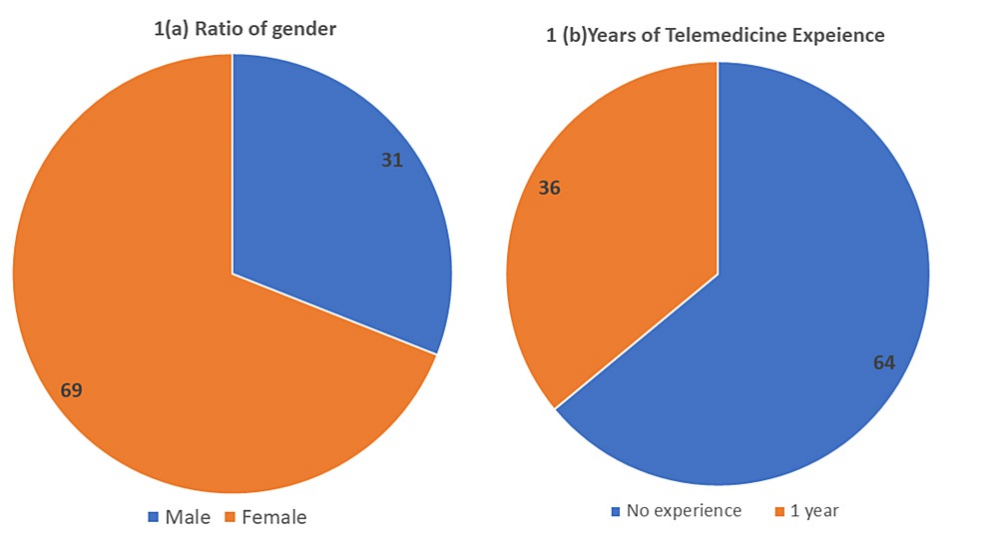
(a) Ratio of gender and (b) years of telemedicine experience

Amongst the total participants of the study (i.e., n=100), 31% are males while 69% are female doctors as shown in Figure 1a, having medical experience of varying years, precisely 22% of the doctors have experienced fewer than five years, 47% of the participants have 5-10 years of experience, 22% have 10-15 years of experience while (n=9) 9% have more than 15 years of experience (Figure 1b), 64% of the participants have no experience of working in telemedicine while only 36% have worked with telemedicine for at least one year.

Moreover, we have observed interesting results regarding the use of modern gadgets by doctors. Among our participants, 51% of the doctors use laptops/mobile phones to search for medical information online, while 37% of them always connect with their patients through social media including WhatsApp and Facebook. However, 73% of doctors have no idea about the legal bindings while interacting with patients online. Also, when asked about the applications of telemedicine technology only 46% of the doctors knew the technology used. While only 42% of the doctors were familiar with telemedicine tools, including a virtual stethoscope, pulse oximeter, etc.

When asked about the importance of the application of telemedicine technology usage for doctors, 28% of the participants agreed that they know about the technology and use this for the cure of patients. Furthermore, the importance of telemedicine usage for patients as a viable approach was agreed upon by 67% of the participants. In this study, 73% of the doctors agreed to the usage of telemedicine for saving money, time, and effort, and 59% of them agreed to the beneficence of telemedicine for patients. We have also compared our results for the experience of doctors using telemedicine with the other significant data shown in Tables 1, 2.

**Table 1:** Comparison of years of telemedicine experience with the quality of care.

Years of Telemedicine Experience	Organizational difficulties while using telemedicine	Technical difficulties while using telemedicine	Overall quality of telemedicine consultations
No experience	60%	62%	64%
1-year experience	40%	38%	36%
Pvalue	0.002*	0.001*	0.001*

**Table 2:** Comparison of years of telemedicine experience with advantages of the use of telemedicine

	No experience		1-year experience		p-value
Perception	Agree	Disagree	Agree	Disagree	
Telemedicine can save time and money	19%	45%	08%	28%	0.012*
Telemedicine is easy to use and navigate	11%	53%	06%	30%	0.001*
Use of telemedicine is beneficial for patients	35%	29%	24%	12%	0.001

Lastly, when the study participants were asked about their views regarding continue using telemedicine, 26% of the participants having no experience with telemedicine agreed that they would like to use telemedicine, 35% agreed to use telemedicine with improvement whereas 3% of them do not like to use the telemedicine technology. Of the participants having one year of experience using telemedicine, 12% of the doctors would like to use telemedicine in the same way, F 23% would like to continue using telemedicine but with improvements whilst only 1% of the doctors would not like to continue using telemedicine services.

## Discussion

In recent times, advances in technology have radically increased the convenience and quality of care that is available digitally [12]. The results of our study show that 64% of the participant doctors had no experience working in telemedicine while 36% had experience, keeping the fact in mind that the present study was conducted in an urban area of the country, the numbers of respondents are lower if we compare it to other countries. All over the world the multitude of telemedicine usage increase tremendously after the pandemic and has now become a permanent fixture. A study conducted by Julia et al. stated that the doximity survey showed the number of physicians working in the USA in the field of telemedicine has doubled from 20% (as of 2020) to 40% (as of 2021) [13]. This shows the increase in the international trend of practicing telehealth [14].

As far as Pakistan is concerned several studies showed that it still lacks the proper system for telemedicine due to which a large number of doctors are still not able to provide their services through this medium. There could be several reasons behind this such as a lack of knowledge and training for telemedicine in medical institutes and hospitals. This was demonstrated in a study conducted by Sana et.al., in Pakistan during the time of COVID-19 to assess the telemedicine knowledge among medical students. The survey showed that medical students should be taught and evaluated about telemedicine so that they will play a significant role in establishing telemedicine services in the healthcare system in the future [15].

However, the results also showed that more than 50% of the doctors use gadgets like mobile phones, and laptops to communicate with their patients using email, social media, and calls. Also, doctors are using these services to keep themselves up to date regarding medical practices, literature reviews, etc. James et al. led a survey in Australia regarding telemedicine technology and found out that doctors have different perspectives and practices to communicate with the patients through internet. As much as social media platforms are becoming common, additional training is required to practice telemedicine online in an ethical manner as it is a difficult task [16]. Another study conducted in the USA demonstrated that younger physicians are practicing telemedicine more than physicians having 5-10 years of experience [17]. The young generation is tech-savvy, and this could be a reason why young doctors are practicing more and using the internet and gadgets to support their patients [18,19].

Conferring to the results of the study that showed over 50% of doctors would like to practice telemedicine with or without improvements in the system on account of the benefits of this technology for both doctors and patients. These results are supported by the study held in India that showed that over 70% of physicians envisioned keeping using telehealth at least occasionally after the pandemic [20]. Another study conducted in China in 2021 supports our results that show physicians are most likely to have increased their telehealth use in the long term [21].

Many participants agreed to the benefits of telemedicine technology for several reasons, including saving money, time, and effort. It is now a known fact that Telemedicine has the potential to decrease healthcare spending by decreasing problems like avoidable emergency department visits, misuse of medications, and prolonged hospitalizations [22,23]. A number of studies support this part of the results. A systemic review exhibited 21 reviews to conclude as telemedicine technology is beneficial from a physician's perspective [24,25].

In the current times, there is an increasing trend of using telehealth technologies in Pakistan in all tertiary care hospitals, because of its efficient and cost-effective means for delivering and accessing quality healthcare services and outcomes. Regarding the organizational and technical difficulties, our results suggest that more than 50% of participants agreed to have organizational and technical difficulties that hinder the proper effective use of telemedicine technology. Organizational factors may be perilous elements of the success of a telemedicine program. Advances in telemedicine technology need to be harmonized with innovations in organizational structure and communication [26,27]. Moreover, workplace support is very important in the proper implementation of telehealth agendas [28,29]. Organizations that are entranced in investing in telemedicine technology support their physicians to adopt the new technology and facilitate its use by making encouraging policies [10,30]. This study is the first of its kind in Pakistan in which we have found out that the telemedicine system is still flawed in Pakistan but needed. Extensive training both in urban and rural areas of Pakistan is needed to get more physicians onboard and help the patients in the right manner. Hence, it is essential to commence more and better eHealth programs on a national level through a constructive cycle of policy, execution, and continuous evaluation.

## Conclusions

The participants of the current study were aware of telemedicine and its advantages; however, despite their agreement majority of doctors emphasized the improvement of the system to deliver better services by using this technology. Furthermore, doctors who were not having experience in telemedicine faced difficulties and had the perception that the technology is not patient-friendly, and they disagreed with telemedicine as a time-saving approach. Studies on larger scales including the doctors of various hospitals (government sector and private sector) and assessment of the perception of patients regarding the telemedicine approach are recommended prior to setting up the grounds for implementation of this advancement in the healthcare system of developing countries.

## References

[1] Haleem A, Javaid M, Singh RP, Suman R (2021). Telemedicine for healthcare: capabilities, features, barriers, and applications. Sens Int.

[2] Flumignan CD, Rocha AP, Pinto AC, Milby KM, Batista MR, Atallah ÁN, Saconato H (2019). What do Cochrane systematic reviews say about telemedicine for healthcare?. Sao Paulo Med J.

[3] López L, Green AR, Tan-McGrory A, King R, Betancourt JR (2011). Bridging the digital divide in health care: the role of health information technology in addressing racial and ethnic disparities. Jt Comm J Qual Patient Saf.

[4] Chunara R, Zhao Y, Chen J, Lawrence K, Testa PA, Nov O, Mann DM (2021). Telemedicine and healthcare disparities: a cohort study in a large healthcare system in New York City during COVID-19. J Am Med Inform Assoc.

[5] Rockwell KL, Gilroy AS (2020). Incorporating telemedicine as part of COVID-19 outbreak response systems. Am J Manag Care.

[6] Albahri AS, Alwan JK, Taha ZK, Ismail SF, Hamid RA, Zaidan A (2021). IoT-based telemedicine for disease prevention and health promotion: state-of-the-Art. J Netw Comput Appl.

[7] Ahmadi M, Meraji M, Mashoof EJ (2018). Rehabilitation. Evidence on telemedicine in Iran-systematic review. J Parasitol Res.

[8] Chellaiyan VG, Nirupama A, Taneja N (2019). Telemedicine in India: where do we stand?. J Family Med Prim Care.

[9] Manchanda S (2020). Telemedicine-getting care to patients closer to home. Am J Respir Crit Care Med.

[10] Mahdi SS, Allana R, Battineni G, Khalid T, Agha D, Khawaja M, Amenta F (2022). The promise of telemedicine in Pakistan: a systematic review. Health Sci Rep.

[11] Nawaz R, Zhou Z, Khalid N (2021). Income-related inequality in distribution of health human resource among districts of Pakistan. BMC Health Serv Res.

[12] Kichloo A, Albosta M, Dettloff K (2020). Telemedicine, the current COVID-19 pandemic and the future: a narrative review and perspectives moving forward in the USA. Fam Med Community Health.

[13] Shaver J (2022). The state of telehealth before and after the COVID-19 pandemic. Prim Care.

[14] Srinivasan R, Wallis KE, Soares N (2022). Global trends in telehealth among clinicians in developmental-behavioral pediatric practice: a COVID-19 snapshot. J Dev Behav Pediatr.

[15] Kazmi S, Yasmin F, Siddiqui SA (2022). Nationwide assessment of knowledge and perception in reinforcing telemedicine in the age of COVID-19 among medical students from Pakistan. Front Public Health.

[16] Brown J, Ryan C, Harris A (2014). How doctors view and use social media: a national survey. J Med Internet Res.

[17] Pierce BS, Perrin PB, Dow AW, Dautovich ND, Rybarczyk BD, Mishra VK (2021). Changes in physician telemedicine use during COVID- 19: effects of practice setting, demographics, training, and organizational policies. Int J Environ Res Public Health.

[18] Koonin LM, Hoots B, Tsang CA, Leroy Z, Farris K, Jolly B (2020). Trends in the use of telehealth during the emergence of the COVID-19 pandemic—United States, January-March 2020. MMWR Morb Mortal Wkly Rep.

[19] Ahmed TJ, Baig M, Bashir MA, Gazzaz ZJ, Butt NS, Khan SA (2021). Knowledge, attitudes, and perceptions related to telemedicine among young doctors and nursing staff at the King Abdul-Aziz University Hospital Jeddah, KSA. Niger J Clin Pract.

[20] Mano MS, Morgan G (2022). Telehealth, social media, patient empowerment, and physician burnout: seeking middle ground. Am Soc Clin Oncol Educ Book.

[21] Callaghan T, McCord C, Washburn D (2022). The changing nature of telehealth use by primary care physicians in the United States. J Prim Care Community Health.

[22] Gajarawala SN, Pelkowski JN (2021). Telehealth benefits and barriers. J Nurse Pract.

[23] Brault ME, Laudermith A, Kroll-Desrosiers A (2022). Telemedicine during COVID-19 response: a welcome shift for younger female healthcare workers. J Gen Intern Med.

[24] Ekeland AG, Bowes A, Flottorp S (2010). Effectiveness of telemedicine: a systematic review of reviews. Int J Med Inform.

[25] Lebedev G, Klimenko H, Fartushniy E, Shaderkin I, Kozhin P, Galitskaya D (2019). Building a telemedicine system for monitoring the health status and supporting the social adaptation of children with autism spectrum disorders. Intelligent Decision Technolog.

[26] Bilal W, Qamar K, Siddiqui A, Kumar P, Essar MY (2022). Digital health and telemedicine in Pakistan: improving maternal healthcare. Ann Med Surg (Lond).

[27] Shen YT, Chen L, Yue WW, Xu HX (2021). Digital technology-based telemedicine for the COVID-19 pandemic. Front Med (Lausanne).

[28] Jennett P, Yeo M, Pauls M, Graham J (2003). Organizational readiness for telemedicine: implications for success and failure. J Telemed Telecare.

[29] Singh J, Albertson A, Sillerud B (2022). Telemedicine during COVID-19 crisis and in post-pandemic/post-vaccine world-historical overview, current utilization, and innovative practices to increase utilization. Healthcare (Basel).

[30] Al-Samarraie H, Ghazal S, Alzahrani AI, Moody L (2020). Telemedicine in Middle Eastern countries: progress, barriers, and policy recommendations. Int J Med Inform.

